# Work environment-related factors and nurses’ health outcomes: a cross-sectional study in Lebanese hospitals

**DOI:** 10.1186/s12912-020-00485-z

**Published:** 2020-10-08

**Authors:** Martine Elbejjani, Mary Abed Al Ahad, Michael Simon, Dietmar Ausserhofer, Nuhad Dumit, Huda Abu-Saad Huijer, Suzanne R. Dhaini

**Affiliations:** 1 Clinical Research Institute, and Department of Internal Medicine, Faculty of Medicine, American University of Beirut, Beirut, Lebanon; 2 Hariri School of Nursing, American University of Beirut, Beirut, Lebanon; 3 Institute of Nursing Science, University of Basel, Basel, Switzerland; 4 Inselspital, Bern, Switzerland; 5 College of Health-Care Professions Claudiana, Bozen, Italy

**Keywords:** nurse’s work environment, Nurses’ health, Nurses’ physical health, Nurses’ mental health, Work environment indicators

## Abstract

**Background:**

Worldwide, studies show a relationship between nurses’ health and some work environment factors; however, data on nurses’ health and self-perceived workload and nursing task allocation are lacking, particularly for Lebanese nurses. We assessed the relationship of several work environment factors: overall workload and specific temporal, physical, mental, effort, frustration, and performance demands (NASA Task Load Index), staffing resources and adequacy and leadership (Practice Environment Scale of Nursing Work Index), teamwork climate (Safety Attitudes Questionnaire), and nursing task allocation (Basel Extent of Rationing of Nursing Care)) with self-reported musculoskeletal, cardiovascular, skin, and mental health diseases (Work Ability Index) and emotional exhaustion (Maslach Burnout Inventory) among Lebanese nurses.

**Methods:**

A cross-sectional self-report survey was distributed to all 289 registered nurses (RNs) in the medical, surgical, and pediatric units in two Lebanese university-affiliated hospitals; 170 RNs had complete data. Adjusted multivariable logistic regression models were used to estimate the association between work environment factors and health outcomes.

**Results:**

The most prevalent outcomes were musculoskeletal disease (69%), emotional exhaustion (59%), and mental health problems (56%); 70% of RNs had ≥2 and 35.29% had ≥4 co-occurring health problems. Musculoskeletal disease was associated with higher overall (OR = 1.36 (95%CI = 1.03, 1.80)), temporal (OR = 1.30 (95%CI = 1.09, 1.55)), and physical demands (OR = 1.20 (95%CI = 1.03, 1.49)), higher task allocation to RNs (OR = 1.11 (95%CI = 1.01, 1.23)) and lower teamwork climate (OR = 0.60 (95%CI = 0.36, 0.98). Higher odds of mental/emotional problems were associated with higher overall, temporal, frustration, and effort demands, and lower teamwork climate, performance satisfaction, and resources adequacy (increased odds ranging from 18 to 88%). Work environment indicators were associated with higher co-occurrence of health problems.

**Conclusions:**

Results show elevated health burden and co-morbidity among Lebanese RNs and highlight the value of comprehensive approaches that can simultaneously improve several work environment factors (namely self-perceived workload, teamwork,, resources, and nursing task allocation) to reduce this burden.

## Background

Worldwide, accumulated data show that nursing is a demanding and stressful profession [1–3]. Nurses work in complex environments, on variable and long rotating shifts, and are exposed to a variety of occupational risks and accidents [4–6]. Empirical research has shown that nurses suffer from physical illness, mental disorder, and emotional exhaustion more than other health practitioners in the general population [7, 8].

Specifically, musculoskeletal diseases are critical occupational health problems among nurses due to the nature of their working conditions and tasks, which involve physical effort and repetitive movements [9, 10]. Research from several countries, including Brazil and Italy [11], Estonia [12], Uganda [13], Nigeria [14], and the United States [15] have reported an annual prevalence of work-related musculoskeletal disorders among nurses ranging between 40 and 85%. Other recurrent health conditions for this occupation are skin diseases, including eczema, allergy, and fungal infections due to exposure to various chemical (e.g., formaldehyde and ethylene oxide), biological (e.g., fungi, viruses, and bacteria), and physical hazards (e.g., radiation, and populous air conditioning) [6, 16]. Some results also suggest that nurses have increased risk of cardiovascular diseases due to rotating shifts and stressful working conditions [17]. In a study conducted in Canadian hospitals, Reed el al. (2018) found that nurses had a high prevalence of several cardiovascular disease risk factors including objectively measured obesity/overweight, hypertension, and high cholesterol levels, as well as self-reported poor mental health, smoking, and diabetes [10]. Another frequently reported health outcomes in this population are mental health problems including anxiety, stress, depression, sleep disorders, and emotional exhaustion [6, 18–20].

In addition to the nature of the nursing tasks – which are largely stress-inducing and put nurses in frequent exposure to the grief and suffering of others – [6, 19, 21, 22], data show that work environment factors in healthcare institutions are associated with nurses’ physical [9, 23, 24] and emotional [21, 22, 25–27] health. Several aspects of the work environment in hospitals have been suggested to influence nurses’ health, including workload demands, nurse managers’ abilities, leadership, staffing resources and adequacy, and the availability of support and teamwork climate [28].

The majority of published studies focus solely on one health outcome or one aspect of the working environment [9, 22]. Data that systematically investigate multiple work environment factors and different nurses’ health outcomes are needed to better characterize how specific components of the work environment influence different health outcomes in this population. Furthermore, to the best of our knowledge, there are no data on how the allocation of nursing tasks (whether performed by registered nurses (RNs) versus other caregivers) relate to nurses’ physical and emotional health; in addition, the integration of detailed self-perceived workload assessments, namely using the domains of the NASA Task Load Index (NASA-TLX) scale [29] is lacking. Nursing task allocation and self-perceived workload are important components of the daily functioning and realities of nurses and could potentially contribute to experiencing both physical and mental health challenges in this population.

In this study, we examined the relationship between various work environment factors and several physical and mental health conditions (musculoskeletal, cardiovascular, skin, mental health disorders and mental exhaustion) among Lebanese nurses. We characterized work environment factors, according to the RICH model (please refer to [30, 31] for details), into structural factors (including leadership, staffing resources and adequacy, and teamwork climate) and process of care factors (self-perceived workload and nursing task performance per professional category) and investigated their relationship to various nurses’ health outcomes while accounting for organizational variables (shift and service). Therefore, this study aimed (1) to describe the prevalence of physical and mental health outcomes among Lebanese nurses and how these outcomes correlate with each other and (2) to assess how different work environment factors relate to specific health outcome and to overall morbidity.

## Methods

### Study design

This study is based on the cross-sectional phase of the RATIONAL study, a longitudinal study on implicit rationing of nursing care among Lebanese patients, described in details elsewhere [31, 32]. The RATIONAL study included two phases of data collection: 1) a cross-sectional baseline phase focused on in-depth assessment of work environment and nurses’ health indicators followed by 2) a longitudinal phase focused on work environment and implicit rationing of nursing care data [31, 32].

### Setting and data collection

The study was conducted in two of the largest university-affiliated hospitals in Lebanon. A cross-sectional survey was distributed to all RNs (*N* = 289) working in the medical, surgical, and pediatrics acute care units in the two selected hospitals.

The participating hospitals include 680 beds in total and allow two shift models: the 8-h (day, evening, and night) and the 12-h shift (day and night).

The 289 RNs were recruited to participate in the study through the nursing management department within each hospital.

Data collection was carried out separately in each participating hospital. In Hospital Center A, a paper version of the study questionnaire was distributed by the head of nursing administration to nurse managers who then distributed it to RNs within each unit; data collection in Center A was conducted in June 2018. In Hospital Center B, data collection occurred in December 2018 through a LimeSurvey electronic version of the questionnaire sent to the RNs’ work-email (obtained from the nursing administration). Differences in data collection procedures (paper versus electronic survey) were based on the request of each hospital’s administration to be in line with their usual and preferred data collection procedures.

The study was approved by the Institutional Review Board of the American University of Beirut on June 1st, 2018 (SBS.2017–0418) and the approval is renewed by the research team on a yearly basis to complete data analysis (The last renewal was obtained on the 17th of December 2019). Each Hospital Center provided written consent and approval to participate and filling the survey was considered as the informed consent of the recruited RNs.

### Sources of data

The study included data from two sources:

1) Hospital administration data which included socio-demographic characteristics of the 289 recruited RNs (gender; age groups: 20–25 yrs., 26-30 yrs., 31–35 yrs., 36–40 yrs., 41–45 yrs., 46–50 yrs., > 50 yrs.; and years of nursing-experience: < 2 yrs., 2–5 yrs., 6–10 yrs., 11–15 yrs., 16–20 yrs., > 20 yrs), collected in an aggregated form as recommended by the American University of Beirut Institutional Review Board to ensure anonymity; and 2) a Nurse Personnel Questionnaire, which integrated several existing instruments (detailed below) to collect data on nurses’ physical and emotional health, nurses’ work environment, allocation of nursing care per professional category, and the type of working shifts and services. The current study and analyses were based on these nurses’ level data.

### Variables and measurements

#### Outcome variables: nurses’ physical and emotional health

*Nurses’ physical and mental health* was measured using four items of the “Work Ability Index (WAI)” which is wildly used in nurses’ health literature and shows high reliability with a Cronbach alpha of 0.78 [31, 33]. The WAI consists of assessing the presence of: 1) musculoskeletal diseases (low back pain, neck and shoulder pain); 2) cardiovascular diseases (heart diseases and hypertension); 3) skin diseases (rash, eczema, allergy); and 4) mental disorders (depression, anxiety, chronic insomnia). Participants self-reported the presence of each disease by selecting one of three answer options: “disease diagnosed by a doctor”, “own diagnosis of the disease”, and “disease does not exist”. We merged the “diagnosis by a doctor” and “own diagnosis” categories as both referred to “the presence of a disease” for each of the outcomes of interest; this resulted in a binary variable for each of the four diseases (0 = absence of the disease; 1 = presence of the disease). In sensitivity analyses, we analyzed the associations of work environment factors with each outcome of interest using the original presence of disease variable with 3 categories (not present, “own diagnosis”, and “diagnosis by a doctor”). Self-reported “own diagnosis” and “diagnosis by a doctor” categories showed overall similar associations with work environment factors, compared to the reference of no disease, in multinomial logistic regression models, which supported our binary classification and merging of the “own diagnosis” and “diagnosis by a doctor” categories.

*Nurses’ work-related emotional exhaustion* was assessed using the “Maslach Burnout Inventory (MBI)” which captures nurses’ feelings experienced at work. The scale has a high reliability (with a Cronbach alpha = 0.90) [31, 34] and consists of nine-items capturing: (1) exhaustion from work, (2) exhaustion at the end of the working day, (3) feelings of fatigue at the start of a working day, (4) fatigue from working all day with human beings, (5) burn-out, (6) frustration, (7) feelings of working too hard, (8) stress from working directly with people, and (9) feelings like being at the end of their rope [34]. The nine items are measured on a 7-point Likert-scale with increasing frequencies; the sum scores range between 0 and 54 with higher scores indicating more frequent emotional exhaustion. In the analysis, the sum scores were used to classify nurses into those experiencing low (scores< 27) and high (scores≥27) emotional exhaustion; the cut-offs were based on a histogram of the sum scores which showed a bimodal distribution separated at the 27 sum value and was in agreement with other reports [35].

#### Work environment factors

##### Self-perceived workload

Self-perceived workload was assessed using the six items of the “NASA Task Load Index (NASA-TLX)” scale (Cronbach alpha = 0.72) [29, 31] which measure mental, physical, and temporal demands as well as frustration, the degree of effort, and performance satisfaction experienced at work. Each of the items was measured on a scale of 0 (low demand) to 10 (high demand). An overall workload score was computed by averaging scores of the six items, with higher scores indicating higher workload demands [29, 36, 37].

#### Leadership and staffing resources and adequacy

Information on leadership and on staffing resources and adequacy were assessed using two subscales with four-items each from the “Practice Environment Scale of the Nursing Work Index (PES-NWI)” questionnaire, given their high reliability (Cronbach alpha 0.84 and 0.74, respectively) and applicability in nursing research [31, 38]. The items were measured using a 4-point Likert scale ranging from one (strongly disagree) to four (strongly agree). We computed an overall leadership score and an overall “staffing resources and adequacy” score, by calculating the unweighted average of the four items within each subscale [39, 40], with higher scores indicating better leadership and staffing resources and adequacy.

#### Teamwork climate

Nurses’ self-perceived teamwork climate was assessed using the teamwork climate subscale from the Safety Attitudes Questionnaire (SAQ) which has a high reliability, with a Cronbach alpha of 0.89 [31, 41]. The subscale includes five items measuring the presence of teamwork and cooperative culture in the workplace using a 5-point Likert scale (1 = strongly disagree, 2 = slightly disagree, 3 = neutral, 4 = slightly agree, 5 = strongly agree); an average score was computed with higher values indicating better teamwork climate [42].

#### Nursing task performance

Participating RNs also completed the “Basel extent of rationing of nursing care (BERNCA)” instrument, which assesses the nurse’s inability to carry out 25 nursing tasks divided into 5 dimensions: 1) activities of daily living (six items), 2) caring and support (ten items), 3) rehabilitation, instruction, and education (four items), 4) monitoring and safety (two items), and 5) documentation (three items) [30]. The BERNCA is internally consistent and homogenous with an interim correlation mean of 0.39, indicating good scale consistency, and a Cronbach alpha of 0.93 [30, 31]. RNs were asked to report whether each of the BERNCA 25 nursing tasks was performed exclusively by RNs = 1, mostly by RNs = 2, mostly by other caregivers = 3, or only by other caregivers = 4. Each of the 25 items was then classified into a binary variable (“exclusively and mostly done by RNs”=1 versus “mostly and only done by other caregivers”=0), the 25 binary items were then summed to compute an overall count for each nurse ranging from 0 (all tasks done either mostly or only by other caregivers) to 25 (all tasks done either exclusively or mostly by RNs).

#### Other work-related measures

The type of shift, service, and hospital center were recorded as categorical variables: type of shift (1 = day shift, 2 = night shift); type of service (1 = medical, 2 = surgical, 3 = pediatrics); and hospital center (1 = Center A, 2 = Center B). Evening shifts were recoded as day shifts due to the small count of evening shift responses.

### Data analysis

The sample’s characteristics were described using means and standard deviations for continuous variables and frequencies and percentages for categorical variables. We then assessed whether the nurses’ health outcomes and work environment factors were different across different types of services and shifts using t-tests, Chi-square tests, and one-way ANOVAs followed by Bonferroni post-hoc comparisons. We assessed the correlation between the various health outcomes using Chi-square tests and Cramer’s V coefficients. We also described the co-occurrence of health problems by counting for each RN, the number of health problems reported (ranging from 0 to 5). We estimated the association of each of the work environment factors and nursing task performance separately with each of the nurses’ physical and mental health outcomes, using multivariable binary logistic regression models adjusting for the type of working shift, service, and hospital center in each model. In additional analyses, we estimated the relationship of each work environment factor with the number of co-occurring diseases. In sensitivity analyses, we repeated these analyses including the explanatory variables of interest in the same model (mutually adjusting for all work environment factors and nursing task performance) and results were largely unchanged. All data analysis was conducted using STATA_14_ and statistical significance was considered at *p*-value < 0.05.

## Results

### Socio-demographic characteristics

The socio-demographic characteristics of the 289 RNs were as follows: Women constituted 73% (*n* = 210) of the sample; 44% (*n* = 127) of RNs were between the age of 20–25 years and 4% (*n* = 10) were older than 45 years. In terms of nursing experience, 43% (*n* = 124) of RNs had 2 to 5 years of experience and 5% (*n* = 14) had more than 20 years of experience. Overall, 189 RNs out of the 289 recruited RNs completed the study questionnaires and 170 (59%) had complete data and thus constituted our analytical sample.

### Description of the study sample and variables

Among the 170 RNs, 67% worked day shifts; 55% worked in the medical units, 25% in the surgical units, and 20% in the pediatrics units (Table 1). The overall self-perceived workload score had a mean of 6.8 out of 10 (SD = 1.3). For specific workload items, the mean scores was of: 8.6 (SD = 1.6) for mental demand, 7.6 (SD = 2.2) for physical demand, 7.6 (SD = 2.0) for temporal demand, 6.5 (SD = 2.4) for frustration, 7.7 (SD = 2.0) for effort, and 7.4 (SD = 2.2) for performance satisfaction (Table 1). The mean scores for the leadership and teamwork scales were 3 (SD = 0.6) out of 4 and 3.8 (SD = 0.7) out of 5, respectively. The indicator for staffing resources and adequacy had a mean of 2.4 (SD = 0.8) out of 4.0 (Table 1). The average count of tasks done by RN computed from the BERNCA score was 16.5 (SD = 3.59) indicating that most necessary nursing tasks were on average done by RNs rather than other caregivers.

**Table 1:** Description of the work environment and health outcomes among participating registered nurses (*n* = 170)

	n (%)	Mean (SD)
Working shift (Day)	114 (67%)	
Type of service		
Medical	94 (55%)	
Surgical	42 (25%)	
Pediatrics	34 (20%)	
Hospital (Center B)	90 (53%)	
**Work environment factors** (scale range)		
^a^Overall workload scale (0–10)		6.8 (1.3)
^a^Workload items (0–10)		
Mental demand		8.6 (1.6)
Physical demand		7.6 (2.2)
Temporal demand		7.6 (2.0)
Frustration demand		6.5 (2.4)
Effort demand		7.7 (2.0)
Performance satisfaction		7.4 (2.2)
^b^Staffing resources adequacy (1–4)		2.4 (0.8)
^b^Leadership (1–4)		3.0 (0.6)
^c^Teamwork climate (1–5)		3.8 (0.7)
^d^Count of tasks performed by RNs (scale range: 0–25)		16.5 (3.6)
**Health outcomes**		
^e^Musculoskeletal disease	117 (69%)	
^e^Cardiovascular disease	63 (37%)	
^e^Skin disease	71 (42%)	
^e^Mental disorder	95 (56%)	
^f^Emotional exhaustion	100 (59%)	

^a^Workload: the overall workload scale is the mean score of the six items of the NASA TLX scale (mental, physical, temporal, frustration, effort and performance satisfaction demands) where each item ranges from 0 to 10; higher scores indicate higher workload demands. ^b^Staffing resources adequacy and leadership: a mean score of staffing resources adequacy subscale (4items) and leadership subscale (4items) ranging from 1(strongly disagree) to 4(strongly agree)

^c^Teamwork climate: a mean score of teamwork subscale (5items) ranging from 1(strongly disagree) to 3(neutral) to 5(strongly agree)

^d^Count of tasks performed by RNs: a score summarizing the number of tasks done exclusively and mostly by RNs

^e^Physical health diseases: 0 = disease does not exist, 1 = disease exists (based on own diagnosis or a diagnosis by doctor). ^f^Emotional exhaustion: 0 = low emotional exhaustion, 1 = high emotional exhaustion

With regards to nurses’ health outcomes, 69% of RNs reported having musculoskeletal diseases, 37% reported having cardiovascular disease, and 42% reported having a skin disease. The prevalence of mental health problems and high emotional exhaustion were 56 and 59%, respectively (Table 1).

### Work environment factors and nurses’ health outcomes across types of shift and service

leadership and teamwork climate as well as five out of the six workload items (mental, physical, and temporal demands, frustration, and effort) were not different between the day and night shifts nor between the medical, surgical, and pediatrics services (Table 2). Observed differences concerned the performance satisfaction measure of the NASA TLX which was lower in the medical (score mean = 7.0, SD = 2.3) versus pediatrics services (score mean = 8.3, SD = 1.9) and nurses’ self-perceived staffing resources and adequacy which was lower in the surgical (subscale score mean = 2.2, SD = 0.9) compared to pediatrics services (subscale score mean = 2.7, SD = 0.8) (Table 2). Nursing task performance did not show variability by shift but was higher in the pediatrics services (mean count of tasks performed by RNs = 18.1, SD = 3.9) as compared to the medical (mean = 16.3, SD = 3.3) and surgical (mean = 15.8, SD = 3.7) services (Table 2).

**Table 2:** Description of the work environment and health outcomes among participating registered nurses (*n* = 170)

	Type of shift		Type of service		
	Day	Night	Medical	Surgical	Pediatrics
	Mean (SD)				
**Work environment factors** (scale range)					
^a^Overall workload scale (0–10)	6.9 (1.3)	6.5 (1.2)	6.8 (1.3)	7.0 (1.1)	6.4 (1.3)
^a^Workload items (0–10)					
Mental demand	8.6 (1.4)	8.4 (1.9)	8.4 (1.6)	8.9 (1.5)	8.6 (1.7)
Physical demand	7.7 (2.2)	7.3 (2.1)	7.5 (2.2)	7.9 (2.2)	7.6 (2.1)
Temporal demand	7.9 (1.8)*	7.2 (2.2)*	7.6 (2.0)	8.1 (1.8)	7.2 (2.1)
Frustration demand	6.7 (2.4)	6.1 (2.4)	6.6 (2.3)	6.6 (2.7)	5.9 (2.3)
Effort demand	7.7 (1.9)	7.7 (2.1)	7.6 (2.0)	8.0 (1.7)	7.5 (2.3)
Performance satisfaction	7.2 (2.3)	7.8 (2.0)	7.0 (2.3)**	7.6 (1.9)	8.3 (1.9)**
^b^Staffing resources adequacy (1–4)	2.4 (0.8)	2.4 (0.9)	2.3 (0.8)	2.2 (0.9)*	2.7 (0.8)*
^b^Leadership (1–4)	3.0 (0.6)	3.0 (0.7)	3.1 (0.6)	2.9 (0.7)	3.0 (0.6)
^c^Teamwork climate (1–5)	3.8 (0.7)	3.8 (0.7)	3.8 (0.7)	3.7 (0.7)	4.0 (0.6)
^d^Count of tasks performed by RNs (scale range: 0–25)	16.6 (0.3)	16.3 (0.5)	16.3 (3.3)*	15.8 (3.7)*	18.1 (3.9)*
	*n (%)*	*n (%)*	*n (%)*	*n (%)*	*n (%)*
**Health outcomes**					
^e^Musculoskeletal disease	84 (74%)	33 (59%)	67 (71%)	30 (71%)	20 (59%)
^e^Cardiovascular disease	46 (40%)	17 (30%)	37 (39%)	12 (29%)	14 (41%)
^e^Mental disorder	69 (61%)	26 (46%)	59 (63%)	19 (45%)	17 (50%)
^e^Skin disease	51 (45%)	20 (36%)	43 (46%)	15 (36%)	13 (38%)
^f^Emotional exhaustion	70 (61%)	30 (54%)	57 (61%)*	29 (69%)*	14 (41%)*

**P-value< 0.01; *P-value< 0.05

^a^Workload: the overall workload scale is the mean score of the six items of the NASA TLX scale (mental, physical, temporal, frustration, effort and performance satisfaction demands) where each item ranges from 0 to 10; higher scores indicate higher workload demands

^b^Staffing resources adequacy and leadership: a mean score of staffing resources adequacy subscale (4items) and leadership subscale (4items) ranging from 1(strongly disagree) to 4(strongly agree)

^c^Teamwork climate: a mean score of teamwork subscale (5items) ranging from 1(strongly disagree) to 3(neutral) to 5(strongly agree)

^d^Count of tasks performed by RNs: a score summarizing the number of tasks done exclusively and mostly by RNs

^e^Physical health diseases: 0 = disease does not exist, 1 = disease exists (based on own diagnosis or a diagnosis by doctor). ^f^Emotional exhaustion: 0 = low emotional exhaustion, 1 = high emotional exhaustion

With respect to health outcomes, only emotional exhaustion was significantly different across the services, with 69% of RNs in the surgical service reporting high emotional exhaustion as compared to 41% in the pediatrics service (Table 2).

### Correlation and co-occurrence of nurses’ health outcomes

The presence of mental health problems was consistently correlated with all reported physical diseases (Cramer’s V ranging from 0.37 with skin disease to 0.45 and 0.46 for musculoskeletal and cardiovascular disease; Table 3A). Other notable correlations included the correlation of cardiovascular and skin disease (Cramer’s V = 0.41) and more moderate correlations between musculoskeletal disease and cardiovascular and skin disease (Cramer’s V ~ 0.25; Table 3A). Only 10% of participating nurses reported no health problems; 70% had more than 2 co-occurring health conditions and over 35% of the sample had 4 or more co-occurring health problems (Table 3B).

**Table 3:** Correlation and co-occurrence of the nurses’ health outcomes (*n* = 170)

**A. Correlation between the nurses’ self-reported physical and mental health outcomes**					
	Musculoskeletal disease	Cardiovascular disease	Mental disorder	Skin disease	Emotional exhaustion
^a^Musculoskeletal	1				
^a^Cardiovascular	0.25**	1			
^a^Mental disorder	0.45**	0.46**	1		
^a^Skin disease	0.26**	0.41**	0.37**	1	
^b^Emotional exhaustion	0.19*	−0.002	0.22**	0.006	1
**B. Co-occurrence of nurses’ health outcomes**					
Number of co-occurring health outcomes			n (%)		
0 (No reported disease)			17 (10.00%)		
1			34 (20.00%)		
2			25 (14.71%)		
3			34 (20.00%)		
4			40 (23.53%)		
5 (all five health outcomes)			20 (11.76%)		

**P-value< 0.01; *P-value< 0.05

^a^Physical health diseases: 0 = disease does not exist, 1 = disease exists (based on own diagnosis or a diagnosis by doctor)

^b^Emotional exhaustion: 0 = low emotional exhaustion, 1 = high emotional exhaustion

### Association of work-related factors with nurses’ reported physical and mental health outcomes

A higher overall workload score was associated with higher odds of musculoskeletal disease (OR = 1.36, 95%CI = 1.03–1.80), mental disorder (OR = 1.53, 95%CI = 1.16–2.01), and emotional exhaustion (OR = 1.88, 95%CI = 1.38–2.55) (Table 4). More specifically, physical demands were related to higher odds of musculoskeletal disease (OR = 1.20, 95%CI = 1.03–1.40), higher temporal demands were associated with higher odds of musculoskeletal and mental health disorders ((OR = 1.30, 95%CI = 1.09–1.55); (OR = 1.24, 95%CI = 1.05–1.47)) and with higher odds of emotional exhaustion (OR = 1.26, 95%CI = 1.06–1.47), higher frustration and lower performance satisfaction were related to higher odds of mental disorders and emotional exhaustion, and higher effort scores were related to higher odds of emotional exhaustion (OR = 1.26, 95%CI = 1.06–1.49) (Table 4).

**Table 4:** Work environment factors and nurses’ health outcomes (*n* = 170)

	Musculoskeletal disease	Cardiovascular disease	Skin disease	Mental disorder	Emotional exhaustion
	OR [95% CI]				
**Work environment factors** (scale range)					
^a^Overall workload scale (0–10)	1.36 [1.03, 1.80]*	1.18 [0.90, 1.53]	1.11 [0.86, 1.43]	1.53 [1.16, 2.01]**^ϵ^	1.88 [1.38, 2.55]** ^ϵ^
^a^Workload items (0–10)					
Mental demand	1.08 [0.87, 1.34]	1.12 [0.90, 1.39]	0.95 [0.77, 1.16]	1.07 [0.87, 1.31]	1.15 [0.94, 1.42]
Physical demand	1.20 [1.03, 1.40]*	1.08 [0.93, 1.25]	1.04 [0.90, 1.21]	1.13 [0.98, 1.31]	1.11 [0.96, 1.29]
Temporal demand	1.30 [1.09, 1.55]** ^ϵ^	1.03 [0.87, 1.21]	1.09 [0.93, 1.29]	1.24 [1.05, 1.47]*^ϵ^	1.26 [1.06, 1.49]**^ϵ^
Frustration demand	1.13 [0.98, 1.30]	1.09 [0.95, 1.25]	1.12 [0.98, 1.29]	1.22 [1.06, 1.41]**^ϵ^	1.39 [1.19, 1.62]**^ϵ^
Effort demand	1.03 [0.87, 1.22]	1.06 [0.91, 1.25]	0.98 [0.84, 1.15]	1.07 [0.91, 1.25]	1.26 [1.06, 1.49]**
Performance satisfaction	1.01 [0.86, 1.18]	0.99 [0.85, 1.14]	1.00 [0.87, 1.16]	0.82 [0.70, 0.97]*	0.81 [0.69, 0.96]*
^b^Staffing resources adequacy (1–4)	0.82 [0.54, 1.25]	1.26 [0.84, 1.90]	1.15 [0.78, 1.72]	0.81 [0.54, 1.20]	0.54 [0.35, 0.82]**^ϵ^
^b^Leadership (1–4)	0.75 [0.43, 1.31]	0.71 [0.42, 1.21]	0.93 [0.56, 1.56]	0.65 [0.38, 1.11]	0.80 [0.47, 1.35]
^c^Teamwork climate (1–5)	0.60 [0.36, 0.98]*	0.81 [0.51, 1.29]	0.71 [0.45, 1.12]	0.62 [0.39, 0.99]*	0.70 [0.44, 1.11]
^d^Count of tasks performed by RNs (0–24)	1.11 [1.01, 1.23]*	1.01 [0.92, 1.11]	1.11 [1.004, 1.23]*	1.07 [0.97, 1.17]	0.94 [0.85, 1.03]

Odds ratios estimated from regression models ran separately for each health outcome and each individual work-related predictor, adjusted for the hospital center (1 = Center A, 2 = Center B), type of shift (1 = Day shift, 2 = Night shift), and type of service (1 = Medical service, 2 = Surgical service, 3 = Pediatric service)

**P-value< 0.01, *P-value< 0.05, ^ϵ^ *P*-value < the false discovery rate corrected significance threshold

^a^Workload: the overall workload scale is the mean score of the six items of the NASA TLX scale (mental, physical, temporal, frustration, effort and performance satisfaction demands) where each item ranges from 0 to 10; higher scores indicate higher workload demands

^b^Staffing resources adequacy and leadership: a mean score of staffing resources adequacy subscale (4items) and leadership subscale (4items) ranging from 1(strongly disagree) to 4(strongly agree)

^c^Teamwork climate: a mean score of teamwork subscale (5items) ranging from 1(strongly disagree) to 3(neutral) to 5(strongly agree)

^d^Count of tasks performed by RNs: a score summarizing the number of tasks done exclusively and mostly by RNs

Better teamwork climate was associated with lower odds for both musculoskeletal (OR = 0.60, 95% CI = 0.36–0.98) and mental health problems (OR = 0.62, 95%CI = 0.39–0.99). Higher staffing resources and adequacy was associated with a 54% lower odds for emotional exhaustion (95% CI =0.35–0.82). A higher count of tasks done by RNs was associated with musculoskeletal and skin disease with each additional task performed by RN associated with a 1.11 higher odds of musculoskeletal (95% CI = 1.01, 1.23) and skin diseases (95% CI = 1.004, 1.23; Table 4).

In additional analyses exploring the number of co-morbidities, we found that higher overall workload, physical and temporal demands, and frustration and lower teamwork climate were associated with a higher number of co-morbidities (Supplementary Table S1).

## Discussion

The data from two Lebanese hospitals show that nurses carry a large health burden with the most prevalent health problems being musculoskeletal and mental conditions and emotional exhaustion. Further, these outcomes showed consistent associations with several work environment factors. Higher total workload and higher temporal demands as well as poorer teamwork climate were related to higher odds of each of these three conditions. All other aspects of workload (except for mental demands) were related to either musculoskeletal disorder or mental health problems and emotional exhaustion. Higher staffing resources and adequacy was associated with lower emotional exhaustion; more frequent performance of nursing tasks by RNs was associated with higher odds of musculoskeletal and skin diseases.

Compared to studies from other countries, the prevalence of health problems among Lebanese nurses tend to be in the higher ranges. For instance, the prevalence of musculoskeletal diseases in our sample was 69% which is closer to the upper range of the reported 40 to 85% prevalence in other countries [11–15]. A recent study conducted by Yang et al. (2019) in China showed that 97% of nurses suffered from at least one work-related musculoskeletal problem with 80.91% reporting low back pain, 78.6% reporting neck pain, and 70.4% reporting shoulder pain [9]. With regards to mental health, reports from around the world have estimated a prevalence of mental health problems among nurses of 45.3% [3] and a prevalence of emotional exhaustion of 26% in Brazil [43], 30% in the USA [19], 43% in Spain [21], and 50% in Turkey [20]. Over half of the RNs in our sample reported the presence of mental disorders and/or high emotional exhaustion, highlighting a higher prevalence of these important problems among Lebanese nurses. In addition to their high prevalence, the presence of mental health problems was strongly correlated with each of the physical diseases outcomes in our sample. This suggests that these problems could result in a potentially important burden not only on emotional wellbeing but also on several other aspects of nurses’ health and function and on the risk of co-morbidities. Indeed, our data show a high prevalence of co-occurrence of health problems among nurses and that only a minority (10%) do not have any health problem at all.

With the exception of temporal demands which are higher in the day shift, we found no variability between the type of working shifts regarding nurses’ self-perceived workload demands, work environment factors, nursing task performance [39], and nurses’ health outcomes. In contrast, recent data have reported higher rates of physical and psychological risks among nurses working in the night shifts compared to the day shifts [6, 44]. Reported staffing resources and adequacy, performance satisfaction, and tasks performed by RNs were higher in pediatric services and emotional exhaustion was lowest in these services. All other work environment factors and health outcomes were not different across pediatric, surgical, and medical services. Overall, the shifts’ and services’ comparisons show large overlap and similarities in work-related factors, self-perceived workload demands, and health outcomes, suggesting that nurses’ subjective experiences and perceptions of their work environment and their role in the care delivery process can be particularly informative and go beyond objectively-measured occupational demands encountered in specific services and shift conditions [45]. These results as well as the associations with health outcomes discussed below highlight the importance of integrating measures such as the NASA TLX self-perceived workload domains to capture nurses’ perceptions and experiences.

Several work-related factors (workload demands, staffing resources and adequacy, team climate, and nursing task performance) were related to musculoskeletal disease and mental health/emotional exhaustion problems. These results suggest potentially more direct and shorter-term relationship between workplace factors and nurses’ musculoskeletal and mental health rather than the potentially longer-term effect on cardiovascular diseases. This notion is further supported by the workload items’ analysis with observed associations between physical demands and musculoskeletal disease specifically whereas workload aspects related to frustration, effort, and performance satisfaction were related to mental health/emotional exhaustion problems. Higher temporal demands and poorer team climate were related to the presence of both musculoskeletal and mental health/emotional exhaustion problems. These findings suggest a close tracking between the perceived problems in workload demands and health outcomes. This could be explained by the cross-sectional nature of the study, wherein a nurses’ perception of their health and work problems could be correlated. Results could also suggest more direct links between different stresses in the environment and their manifestation in a related health problem among nurses.

Previous studies looking at specific work environment factors showed that higher workload demand and the absence of teamwork climate were significantly associated with the presence of musculoskeletal diseases among RNs [9, 24]. A cross-sectional study in Spain showed a negative relationship between emotional exhaustion and staffing resources and adequacy, nurse manager ability, leadership, and support climate [21]. Furthermore, teamwork climate manifested in good working relationships and communication between nurses was significantly associated with lower emotional exhaustion among nurses working in Brazilian [46] and Italian hospitals [22]. Our findings complement these results and further suggest that several aspects of the working environment are related to musculoskeletal and mental/emotional health among nurses with more direct relationships between certain physical-related demands and musculoskeletal disease whereas associations with mental health problems and mental health exhaustion is widespread across 7 of the 10 investigated work-related factors.

In addition, a novel finding is the inverse relationship between higher performance of nursing tasks by RNs and higher odds of musculoskeletal and skin disease. Nursing task performance was not related to the mental health outcomes, suggesting that this component of the work may have a more direct impact related to physical demands and problems (e.g., more daily tasks, more usage of gloves, higher exposure to strain and skin infections) and may not contribute substantially to the general work-related stress that can influence mental health.

## Limitations

The study findings have to be interpreted in the light of several limitations. The study might have included a response bias which is a common phenomenon in healthcare research where self-reported data is used [47]. The surveys were all filled without identifying information, yet the role of social desirability and potentially biased rating of self-assessed behavior cannot be ruled out. In addition, the survey was cross-sectional so having a disease could be related to more negative views on the working environment. Another limitation is that the current study did not adjust for the socio-demographic characteristics of the RNs in the regression models due to institutional and ethical considerations, which provided the socio-demographics in an aggregated manner to avoid identifying individual RNs. We recruited all RNs working in the three units of the two participating hospitals, but the proportion of respondents with complete data was 58.8%, which could have resulted in a potentially selective sample. Further, this might have introduced some restrictions in the detection of associations of smaller magnitude and associations with leadership and staffing indicators as per our power calculations. The study involved multiple hypothesis testing and spurious associations could have been observed. However, there were consistencies in the direction of the associations and their magnitude. After correction for multiple testing using false discovery rate under dependence assumptions using the Benjamini and Yekutieli [48] methods, associations of total workload scores, temporal demand, frustration, and staffing resources and adequacy with mental health and emotional exhaustion and that of temporal demand with musculoskeletal disease remained statistically significant; and conclusions were in line with the wider pattern of associations of work-related factors and mental health/emotional exhaustion problems. Lastly, our results focused on two large private hospitals in an urban setting; more data are needed to characterize the experiences of nurses in other public and more rural settings in Lebanon and the region.

## Conclusions and research and nursing management implications

Our study revealed a high prevalence of health problems and co-morbidity among registered Lebanese nurses, with the most prevalent conditions being musculoskeletal and mental/emotional health conditions. Our results showed that several work-related factors were associated with these health conditions. Overall workload, temporal demands and team climate were related to both these health outcomes. In addition, results revealed a more direct association between musculoskeletal and skin disorders and factors related to physical burden, namely higher physical and higher allocation of nursing tasks to nurses, whereas mental health problems were related to various components of workload demands, staffing resources and adequacy, and teamwork climate.

Our findings motivate future research and efforts to optimize nursing task allocation and performance in a manner that can simultaneously protect patients’ care and safeguard nurses’ health. From a local public health perspective, results highlight high health risk among Lebanese nurses, particularly mental and emotional health risks and suggest the value of more comprehensive strategies that can improve several work environment realities, including staffing resources, workload, and team climate.

In addition, future studies will benefit from assessing more detailed aspects of nurses’ health, including identifying the prevalence of specific types of health problems within each health category (e.g., shoulder pain, depression, etc.) as well as risk factors for chronic diseases (e.g., hypertension, diet, earlier markers of emotional distress). Such data can help in better identifying mechanisms by which certain work environment factors influence health and in improving the timely diagnosis, course, and management of health burden among nurses.

## Supplementary information

**Additional file 1: Supplementary Table S1.** Work environment factors and the co-occurrence of health problems among registered nurses.

## Data Availability

The datasets generated and analyzed for the current study are not publicly available due to IRB agreements but are available from the corresponding author on reasonable request.

## Abbreviations

RN: Registered nurse
MBI: Maslach Burnout Inventory scale
NASA-TLX: NASA Task Load Index scale
PES-NWI: Practice Environment Scale of the Nursing Work Index questionnaire
SAQ: Safety Attitudes Questionnaire
BERNCA: Basel extent of rationing of nursing care
