# Supplementary material for: Work environment-related factors and nurses’ health outcomes: a cross-sectional study in Lebanese hospitals
Source: BMC Nurs. 2020 Oct 8;19:95. doi: 10.1186/s12912-020-00485-z (PMC7545948; doi:10.1186/s12912-020-00485-z)
Supplement: Supplementary file 1 — Additional file 1: Supplementary Table S1. Work environment factors and the co-occurrence of health problems among registered nurses. [file 12912_2020_485_MOESM1_ESM.docx]

**Supplemental Table S1. Work environment factors and the co-occurrence of health problems among registered nurses (n=170)**

|  | Health conditions  β [95%CI] |
| --- | --- |
|  |  |
| Overall Workload | 0.35 [0.17, 0.54]** |
| Mental demand | 0.08 [-0.08, 0.23] |
| Physical demand | 0.12 [0.01, 0.23]* |
| Temporal demand | 0.19 [0.07, 0.31]** |
| Frustration demand | 0.19 [0.09, 0.28]** |
| Effort demand | 0.08 [-0.04, 0.20] |
| Performance satisfaction | -0.09 [-0.20, 0.02] |
| Staffing and resource adequacy | -0.14 [-0.44, 0.16] |
| Leadership | -0.30 [-0.69, 0.08] |
| Teamwork climate | -0.42 [ -0.76, -0.09]* |
| Count of tasks performed by RNs (0-25) | 0.05 [ -0.02, 0.11] |

**P-value<0.01, *P-value<0.05

The model is run as a linear regression.

All the models ran separately and were adjusted for shift, service, and hospital.
